# Elektive Tracheostomie bei COVID-19-Patienten – Erfahrungen mit einem standardisierten interdisziplinären Vorgehen

**DOI:** 10.1007/s00106-020-00917-x

**Published:** 2020-08-25

**Authors:** A. Pudszuhn, S. Voegeler, C. Berger, S. Treskatsch, S. Angermair, S. Hansen, V. M. Hofmann

**Affiliations:** 1 Klinik für Hals‑, Nasen‑, Ohrenheilkunde, Campus Benjamin Franklin, Charité Universitätsmedizin Berlin, Hindenburgdamm 30, 12203 Berlin, Deutschland; 2 Klinik für Anästhesiologie und Intensivmedizin, Campus Benjamin Franklin, Charité Universitätsmedizin Berlin, Berlin, Deutschland; 3 Institut für Hygiene und Umweltmedizin, Campus Benjamin Franklin, Charité Universitätsmedizin Berlin, Berlin, Deutschland

**Keywords:** Tracheotomie, SARS-CoV‑2, Medizinisches Personal, SOP, Coronavirus, Tracheotomy, SARS-CoV‑2, Healthcare staff, SOP, Coronavirus

## Abstract

Berichtet wird über die Erfahrungen mit einer interdisziplinären klinikinternen SOP (Standard Operation Procedure) zur Tracheostomie (TS) bei „Coronavirus-Disease“(COVID-19)-Patienten, unter Berücksichtigung der allgemeinen nationalen und internationalen Empfehlungen. Der interdisziplinär festgelegte operative Zeitpunkt der TS aufgrund einer prolongierten invasiven Beatmung und frustranen Weaning-Versuchen betraf Phasen sowohl hoher als auch niedriger Erkrankungsaktivität. Es wurden 5 TS bei Patienten mit einem Durchschnittsalter von 70,6 Jahren durchgeführt. Neben den Standard-COVID-19-Schutzmaßnahmen für das medizinische Personal zur Vermeidung einer nosokomialen COVID-19-Infektion führt die SOP-unterstützte Kommunikation während der TS zu einer periprozeduralen Sicherheit aller Beteiligten. COVID-19-Erkrankungen des medizinischen Personals der beteiligten Abteilungen sind bisher nicht bekannt.

## Abstract (en)

Experience with an interdisciplinary SOP (standard operating procedure) for tracheostomy (TS) in COVID-19 patients, taking into account the general national and international recommendations, is reported. The operative timing of TS due to prolonged invasive ventilation and frustrating weaning attempts was determined on an interdisciplinary level and involved phases of both high and low disease activity. Five TS were performed in patients with an average age of 70.6 years. In addition to the standard COVID-19 protective measures for medical staff to avoid nosocomial COVID-19 infection, SOP-supported communication during the TS leads to periprocedural safety for all involved. COVID-19 infections among medical staff in the departments involved are not yet known.

## Hintergrund

Die von der Weltgesundheitsorganisation (WHO) seit dem 11.03.2020 als Pandemie eingestufte Infektion mit dem Severe acute respiratory syndrome-Coronavirus‑2 (SARS-CoV-2) und der Erkrankung Corona Virus Disease 2019 (COVID-19) zählt weltweit über 6 Mio. bestätigte Fälle und 371.166 Todesfälle (Stand 01. Juni 2020; [3]). Das Robert-Koch-Institut (RKI) kann zwar von der Meldewoche 17 zu 18 einen Rückgang der Infektionszahlen um 41 % in Deutschland verzeichnen, jedoch ist nach Lockerung der initialen Maßnahmen zur Eindämmung des Infektionsgeschehens wieder mit steigenden Infektionszahlen zu rechnen [27]. Aufgrund der nicht in absehbarer Zeit zu erreichenden Herdenimmunität und derzeit nur experimenteller medikamentöser Behandlungsmöglichkeit bzw. fehlender präventiver Maßnahmen durch flächendeckende Impfungen erfordert die Pandemie somit das Management langzeitbeatmeter COVID-19-Patienten [14].

Im Vergleich zu SARS-Infektionen 2002 und vermutlich dem Middle East Respiratory Syndrom (MERS) 2012 erfolgt die Mensch-zu-Mensch des SARS-CoV-2-Übertragung durch Tröpfcheninfektionen besonders leicht, vorrangig durch Tröpfchen (>5 µm) möglicherweise auch durch Tröpfchenkerne (<5 µm; sog. luftgetragene Übertragung) und Kontaktübertragung [22, 40]. Insbesondere im medizinischen Bereich ist in der Umgebung von COVID-19-Patienten eine Übertragung durch mit respiratorischen Sekreten kontaminierte Oberflächen nicht auszuschließen [25, 40]. Daraus abgeleitet sind die Schutzmaßnahmen für das medizinische Personal: neben der Händehygiene nach WHO-Indikation, die Nutzung einer persönlichen Schutzausrüstung (PSA) bestehend aus einem wasserundurchlässigen Schutzkittel, einem Mund-Nasen-Schutz, keimarmen Handschuhen und ggf. einer Schutzbrille bei einem Kontakt mit COVID-19-Patienten oder COVID-19-Verdachtsfällen ohne eine Aerosolbildung indiziert. Bei allen Behandlungen und Eingriffen, die mit einer Aerosolbildung einhergehen, ist mindestens eine FFP(„filtering face piece“)-Maske und ein Augenschutz in Form einer Schutzbrille oder eines Faceshields zu tragen [2, 28]. Die konsequente Nutzung dieser Barrieremaßnahmen ist besonders in den Fachrichtungen (z. B. HNO, Anästhesie) angezeigt, in denen aufgrund der Manipulation an den Atemwegen mit möglicher Aerosolbildung ein erhöhtes Expositionsrisiko für das medizinische Personal besteht.

Bereits bei MERS- und SARS-Infektionen und in den sehr stark von der Pandemie mit COVID-19 betroffenen Ländern wurde auf ein besonderes Infektionsrisiko für medizinisches Personal atemwegsnaher Fachrichtungen insbesondere bei Aerosol-induzierenden Maßnahmen hingewiesen [11, 23]. Daher ist die konsequente Prävention nosokomialer COVID-19-Infektionen von Mitarbeitern im Gesundheitswesen von entscheidender Bedeutung. Gemäß der Mitteilung des italienischen Chirurgenverbandes FNOMCEO (Federazione Nazionale degli Ordini dei Medici Chirurghi e degli Odontoiatri) sind bis zum 22. April 2020 infolge der COVID-19-Pandemie 151 Ärzte in Italien gestorben [39]. Bei einer zu diesem Zeitpunkt dokumentierten Gesamtzahl von 26.977 Todesfällen durch COVID-19 entspricht das einem Anteil an Ärzten von 0,56 % (weiteres medizinisches Personal ausgenommen). Ende März 2020 lag der Gipfel der Infektionsrate beispielsweise in Italien in den Gesundheitsberufen bei etwa 10 % mit deutlichen regionalen Unterschieden, wobei die Infektionsquelle möglicherweise nicht nur von Patienten ableitbar ist [5].

Die meist notwendige invasive Langzeitbeatmung schwerstkranker COVID-19-Patienten kann im Verlauf die elektive Anlage einer Tracheostomie (TS) erfordern [14]. Dieser Eingriff birgt durch die Möglichkeit der virushaltigen Aerosolbildung ein besonders hohes Risiko der SARS-CoV-2-Exposition für das medizinische Personal [14, 28, 38]. Aufgrund der individuell unterschiedlichen Erkrankungsverläufe ist der Zeitpunkt einer TS nicht immer in einer Phase der geringeren COVID-19-Erkrankungsaktivität oder erst nach erfolgtem Ausschluss fortbestehender Viruslast planbar. Deshalb kommt neben den genannten Schutzmaßnahmen der Risikominimierung bzw. kontrollierten Freisetzung von potenziell infektiösem Aerosol eine entscheidende Rolle zu [21, 36].

Orientierend an den bekannten Daten der SARS-Epidemie haben mehrere nationale HNO-Gesellschaften bereits Empfehlungen zur TS bei COVID-19 ausgesprochen [13, 14, 16, 18, 21, 30, 32, 33]. Die publizierten Guidelines und Protokolle zeigen jedoch auch unterschiedliche Standards [4, 10, 14, 17, 24, 35]. Zur Risikominderung im Rahmen der COVID-19-Pandemie ist es zusätzlich zu diesen Empfehlungen wichtig, eine lokale, gut strukturierte und an die Ressourcen der Klinik angepasste Verfahrensweise zu erstellen.

## Standardisiertes Vorgehen

Durch ein standardisiertes Vorgehen bei einer elektiven TS von COVID-19-Patienten im Sinne einer Standard Operating Procedure (SOP), wurde ein Ablauf festgelegt, der insbesondere im Bereich kritischer Schritte mit potenziellen Auswirkungen auf die Gesundheit des medizinischen Personals präventive Maßnahmen bestimmt. Diese SOP legt detailliert die prozedurspezifischen Erfordernisse unter Berücksichtigung der klinikinternen Gegebenheiten fest und wird durch die beteiligten Disziplinen konsentiert. Außerdem muss diese SOP bedarfsabhängig einer regelmäßigen Prüfung unterliegen. Im Fall der SOP zur TS bei COVID-19-Patienten sind in diesen Prozess die operativen Fachrichtungen, die Anästhesiologie/Intensivmedizin, das operative als auch anästhesiologische Pflegepersonal und die Krankenhaushygiene einbezogen.

Klinikintern wurde die offene TS in tiefer Narkose und Vollrelaxation als Methode der Wahl favorisiert, da sie sowohl den Vorteil einer verkürzten und kontrollierten Aerosolexpositionszeit und -menge als auch die Platzierung großlumiger Trachealkanülen (TK) bietet. Im Fall von SARS-CoV-2-positiven Patienten ist sie der dilatativen Tracheotomie aufgrund der Möglichkeit zur chirurgischen Blutstillung (Patienten mit Antikoagulation), längeren Apnoezeiten, häufigeren Diskonnektionen der Beatmung und dauerhaft erforderlicher bronchoskopischer Kontrolle vorzuziehen [8, 37].

Des Weiteren wurde klinikintern festgelegt, dass eine TS bei COVID-19-Patienten nur im Operationssaal durchgeführt wird, um die Vorteile der dortigen Raumlufttechnik zur Abführung der z. B. durch Aerosole belasteten Luft, der optimalen Lagerung des Patienten und der uneingeschränkten Positionierung des Operationsequipment zu nutzen [20]. Die zuführenden Operationssaaltüren werden für den Zeitraum der Operation allseitig mit dem Warnhinweis auf die Behandlung eines COVID-19-Patienten beschildert und bleiben durchgehend während der Operation geschlossen. Nicht benötigtes Equipment wird aus dem Operationssaal entfernt. Grundsätzlich sind TS auf Intensivstation (ITS) auch möglich, jedoch ist derzeit ungeklärt, ob sie Vorteile bezüglich eines geringeren Infektionsrisikos bringen [14].

Zusätzlich zur empfohlenen sterilen flüssigkeitsundurchlässigen langärmligen Operationsbereich-Schutzkleidung und sterilen Operationshandschuhen tragen alle am Eingriff Beteiligten entsprechend der WHO-Empfehlungen Gesichtsschutz (immer Faceshield) und mindestens eine FFP2- oder -3-Maske ohne Ausatemventil als PSA [2, 20]. Die Effektivität der Infektionsschutzes ist entscheidend vom Test eines korrekten Maskensitzes („fitting test“) abhängig [14]. Ist dies anatomisch nicht möglich, sollte die TS durch einen anderen Operateur erfolgen. Eine gegenseitige Kontrolle der korrekten Anlage aber auch der postprozeduralen Ablage der PSA inklusive der Händedesinfektion nach Berühren des Kittels und der Maske sowie vor möglichen Gesichtskontakten im Sinne eines „buddy check“ wird empfohlen. Es ist empfehlenswert, die konkreten Abläufe durch eine externe Supervision der Krankenhaushygiene zu prüfen, um mögliche Lücken in der Umsetzung identifizieren zu können [15].

Um ein sicheres operatives und anästhesiologisches Management zu ermöglichen und das Risiko einer Infektion des medizinischen Personals zu minimieren, werden nur erfahrene Anästhesisten und HNO-Operateure sowie im Umgang mit der SOP geschultes Personal eingesetzt. Zum Team gehören immer 6 Personen (Anästhesist, Anästhesiepflege, Operateur, Operationsassistenz, Operationspflege steril und unsteril).

Zur Durchführung einer elektiven TS bei SARS-CoV-2-positiven Patienten ist eine interdisziplinäre Planungsphase erforderlich, welche prä-, intra- und postoperative Erfordernisse erfasst.

### Präoperative Phase

Der Zeitraum dieser Phase kann mehrere Tage umfassen. Bei der Indikationsstellung einer TS unter COVID-19 spielt der Aktivitätsstatus der Erkrankung eine wesentliche Rolle, um das Risiko einer nosokomialen Infektion der Beteiligten zu reduzieren [14]. Aus anästhesiologischer Sicht ist insbesondere die Einschätzung der Apnoetoleranz zur hinreichenden Oxygenierung neben der grundsätzlichen klinischen Stabilität des Patienten für die Narkose entscheidend. Die erforderlichen anästhesiologischen und operativen Maßnahmen einschließlich Apnoephasen müssen sicher toleriert werden. Zudem können verschiedene Parameter (schwierige Re-Intubation bei Tubusdislokation, Druckläsionen durch den Tubus in Bauchlage, Cuff-Dichtigkeitsprobleme) die Indikation zur TS beeinflussen. Es sollte ein möglichst günstiger Zeitpunkt der bereits in Deeskalation befindlichen Beatmungstherapie vorliegen. Der HNO-Operateur muss präoperativ insbesondere Nebenerkrankungen (Gerinnungsstörungen durch Hämodialyse, Antikoagulation, Thrombozytopenie, Anämie) und die anatomischen Besonderheiten beachten (Adipositas, Schilddrüsenoperationen, Reklinationsmöglichkeiten, Bildgebung), um intraoperativ ein sicheres und rasches Vorgehen zu ermöglichen. Da thrombembolische Ereignisse bei COVID-19 erhöht sind, wird eine moderate Antikoagulation empfohlen [19]. Sowohl durch Sepsis und Hämodialyse als auch extrakorporale Membranoxygenierung (ECMO) sind Gerinnungsstörungen bei COVID-19-Erkrankten aggraviert [14]. Die Koagulopathien, wie Thrombozytopenie, Verlängerung der Blutungszeit durch Antikoagulation, sollten für den Operationszeitpunkt optimiert werden, um intraoperative Blutungskomplikationen zu reduzieren. Auf Grundlage dieser Informationen werden Indikation und Zeitpunkt des Eingriffs gemeinsam interdisziplinär festgelegt.

**Figure 1:**
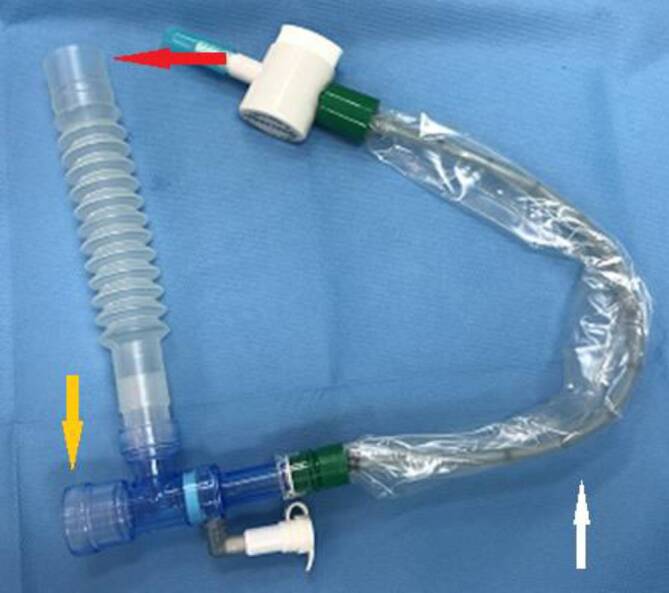

Für alle Beteiligten muss ausreichend PSA vorhanden sein. Alle erforderlichen Operationsmaterialen (Tracheostomiesieb, TK einschließlich Ersatz in verschiedenen Größen, virendichte Absaugfilter für Rauchabsaugung, PSA, sterile Abdeckung) werden in einem Container dauerhaft deponiert und sind so auch in Notfällen griffbereit. Zur Konnektion an die TK müssen die erforderlichen Anteile des Beatmungssystems („head and moisture exchange filter“, HME-Filter, geschlossenes Absaugsystem, Winkelstück und Tubusverlängerung) sowie ein individuell festgelegter Endotrachealtubus (ET) für den Fall einer Cuff-Beschädigung vorhanden sein (Abb. 1).

Es werden entsprechend intensivmedizinischer Standards blockbare, ungefensterte TK mit subglottischer Absaugung gewählt, um eine dauerhafte peristomale Sekretreduktion während der fortgesetzten Beatmungstherapie zu erreichen [1].

### Intraoperative Phase

Zum maßvollen Umgang mit Zeit- und Material(PSA)-Ressourcen wird der Patient von der ITS direkt durch das erfahrene und speziell für COVID-19-Patienten geschulte anästhesiologische COVID-Airway-Team in den Operationssaal transferiert.

**Figure 2:**
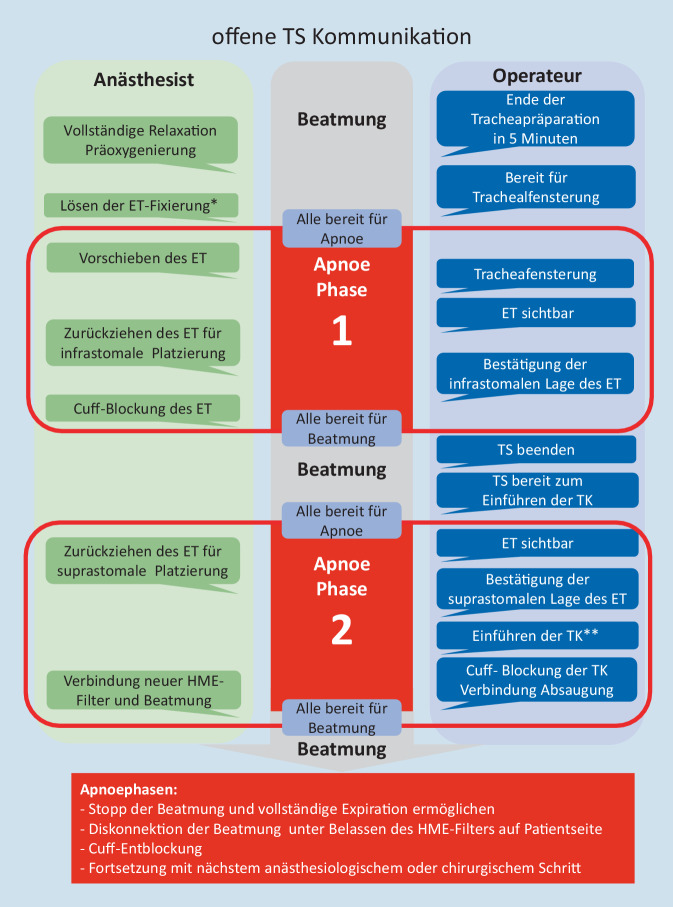

Nach sorgfältiger Umbettung und Lagerung des Patienten erfolgt gemäß WHO-Checkliste das Team-Time-Out, in dem neben den speziellen COVID-19-Infektion weitere patientenbezogene Besonderheiten Erwähnung finden. Intraoperativ regelt ein klarer Workflow die Abläufe. Der gesamte Eingriff wird in ausreichend tiefer Sedierung und Relaxierung unter Neuro- und Relaxationsmonitoring („train of four“: TOF = 0) durch den Anästhesisten durchgeführt. Vor jeder potenziell aerosolfreisetzenden Maßnahme erfolgt eine unmissverständliche Kommunikation über den Operationsfortschritt und relevante anästhesiologische Maßnahmen in Zusammenhang mit der Beatmung (Abb. 2). Es ist zu beachten, dass die Verständigung aufgrund der FFP-Masken und dem Faceshield erschwert sein kann. Die Operationsschritte, wie das Vorschieben des ET, die Inzision der Trachea sowie das Einführen der TK, müssen immer nach standardisierter Vorbereitung des Patienten und höchster Aufmerksamkeit aller Beteiligten in Apnoe erfolgen. Dabei ist insbesondere die interdisziplinäre Interaktion bei der Inzision der Trachea entscheidend. Eine Cuff-Beschädigung ist zwingend zu vermeiden. Ohne abgedichtete Trachea sollte keine Beatmung erfolgen, um eine Gefährdung des Personals durch Exposition virushaltiger Aerosolbildung zu vermeiden. Ein verlängertes Aussetzen der Beatmung hingegen resultiert in Oxygenierungsproblemen und gefährdet damit die Patientensicherheit. Zur Vermeidung einer Cuff-Beschädigung sind exakte Schritte in der SOP formuliert (Abb. 2). Sollte der Cuff jedoch dennoch beschädigt werden, ist eine sofortige transstomale Sicherung des Atemwegs mit dem vorab bereitliegenden ET durch den Operateur möglich, um eine für das Personal gefahrlose und den Patienten angepasste Beatmung zu beginnen. Wenn operationstechnisch möglich, wird die TS bei liegendem transstomalen ET beendet. Alternativ kann durch den Anästhesisten unter Fortsetzung der Beatmung und stabilen Verhältnissen z. B. über einen Wechselstab ein neuer transglottischer ET platziert und die TS kann beendet werden.

Die Blutstillung sollte möglichst durch Unterbindungen erfolgen. Bei Kauterisierung wird eine permanente Absaugung mit einem speziellen virendichten Rauchpartikelfiltersystem durchgeführt (Richtlinien und klinische Untersuchungen zu COVID-19 ausstehend). Die tracheokutane Anastomose wird mit resorbierbarem Nahtmaterial (Polydioxanone) verschlossen. Die Trachealkanülenfixierung erfolgt zusätzlich per nichresorbierbarer Naht, um eine Dislokation bei Beatmungen in Bauchlage zu vermeiden.

In Apnoe erfolgt nach Einführung und Blockung der TK die Konnektion des bereitliegenden Winkelstücks mit dem geschlossenen Absaugsystem und der Tubusverlängerung einschließlich neuem HME-Filter (Abb. 1). Erst danach kann der mit dem ET verbundene HME-Filter patientenfern diskonnektiert und mit dem neuen HME-Filter verbunden werden. Nach offener Kommunikation „Alle bereit für Atmung“ wird erneut mit der Beatmung begonnen (Abb. 2). Absaugungen von Sekret der Mundhöhle erfolgen erst nach platzierter und geblockter Trachealkanüle.

Postoperativ wird der Patient direkt vom intraoperativ betreuenden anästhesiologischen Team auf die ITS zurückverlegt.

### Postoperative Phase

Der erste Trachealkanülenwechsel sollte, insbesondere bei aktiver COVID-19-Erkrankung, zunächst so spät wie möglich erfolgen. Cuff-Leckage, Blutungen, Kanülenverlegungen oder Wundheilungsstörungen können einen frühzeitigeren Wechsel erforderlich machen. In jedem Fall müssen auch beim Wechsel der Kanüle alle Schutz- und Präventionsmaßnahmen sowie Kommunikationsabläufe (Abb. 2), im Sinne der Mitarbeiter- und Patientensicherheit beachtet werden.

## Erfahrungen mit Tracheostomien von COVID-19-Patienten

Seit Einführung der SOP wurden 5 Patienten (1 weiblich, 4 männlich) mit COVID-19-Erkrankung tracheostomiert (Tab. 1). In allen Fällen erfolgten präoperativ eine interdisziplinäre Einschätzung der Indikation und die Festlegung des optimalen Operationszeitpunktes aus anästhesiologischer und operativer Sicht. Das Durchschnittsalter der Patienten beträgt 70,6 Jahre (Spannweite 57–77 Jahre). Bei allen 5 Patienten war zum Operationszeitpunkt noch SARS-CoV-2-RNA in der PCR aus dem tiefen (bronchoalveoläre Lavage/Tracheobronchialsekret) und oberen Respirationstrakt (Nasen-Rachen-Abstrich) nachweisbar. Alle Patienten hatten eine Anämie und eine Koagulopathie, 2 Patienten eine Thrombozytopenie. Der durchschnittliche Body-Mass-Index (BMI) betrug 26,3 kg/m^2^, 3 Patienten wiesen eine Präadipositas auf, ein Patient eine Adipositas Grad I. Alle Patienten hatten mindestens eine pulmonale bzw. kardiovaskuläre Vorerkrankung. In 3 Fällen waren anamnestisch operative Eingriffe am Tag (Fall 2: Iliacalaneurysmaresektion) bzw. in den Wochen (Fall 4/5: popliteocruraler Venenbypass, Perkutane transluminale Koronarangioplastie mit Stenteinlage) vor der stationären Aufnahme als COVID-19-Patienten vorausgegangen.

**Table 1:** 

Patient	Alter (J) m/w	Indikation zur Tracheostomie	Nebendiagnosen	BMI kg/m^2^	Hb g/dl	Thrombozyten/nl	PTT s
1	57 W	Hohe Beatmungsdrücke, ausgeprägte Druckläsionen Mundwinkel und Zungenschwellung durch ET	Asthma bronchiale Bipolare Störung Z. n. Schilddrüsenresektion	30,8	8,1	96	40,2
2	76 M	Prolongierte ET-Intubation Frustranes Weaning	Iliacalaneurysma interna rechts Z. n. Aneurysmaresektion mit Patchplastik Iliaca communis rechts Arterielle Hypertonie Koronare 3‑Gefäß-Erkrankung, Z. n. Stent	26,7	8,5	284	44,3
3	67 M	Wiederholtes Beatmungsleck, Beatmung in Bauchlage eingeschränkt, ECMO	Lungenarterienembolie bei tiefer Beinvenenthrombose links akutes Nierenversagen, Sulcale Blutauflagerungen rechts frontal und parietal	25,7	9,2	42	50
4	77 M	Prolongierte ET-Intubation Frustranes Weaning	Koronare Herzerkrankung Z. n. PTCA und DE-Stenting Diabetes mellitus Typ II Spinalkanalstenose Z. n. Dekompressionsoperation Z.n. Hüfttotalendoprothese	29,1	7,7	249	53
5	76 M	Prolongierte ET-Intubation Frustranes Weaning	Peripherer arterieller Verschlusskrankheit Stadium IV nach Fontaine, Z. n. popliteocruralem Venenbypass Paroxysmales VHF Schrittmacher bei Sick-Sinus-Syndrom Arterielle Hypertonie	19,4	9,1	302	56,5

*m* männlich, *w* weiblich, *BMI* Body-Mass-Index, *Hb* Hämoglobin, *PTT* Partielle Thromboplastinzeit, *J* Jahre, *ET* Endotrachealtubus, *ECMO* extrakorporale Membranoxygenierung, *PTCA* Perkutane transluminale Koronarangioplastie, *DE-Stenting* „Drug-eluting-Stenting“

**Figure 3:**
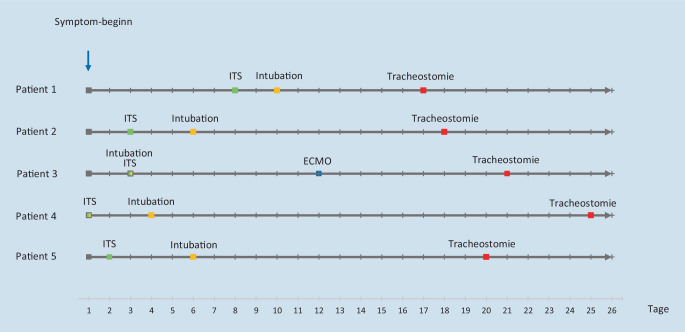

Die Erkrankungsverläufe der Patienten zeigen eine mittlere Zeitspanne vom Beginn der Symptome bis zur erforderlichen intensivmedizinischen Überwachung von durchschnittlich 3 Tagen (Spannweite 1–7 Tage, Ausschluss Patient 4, anamnestisch aufgrund deliranter Symptomatik bei ITS-Aufnahme zu erheben; Abb. 3). Aufgrund der zunehmenden respiratorischen Insuffizienz dauerte es nach Aufnahme auf der ITS bis zur Intubation durchschnittlich 2,8 Tage (Spannweite 2–4 Tage). Der Zeitraum von der Intubation bis zur operativen TS betrug durchschnittlich 13 Tage (Spannweite 7–21 Tage).

**Figure 4:**
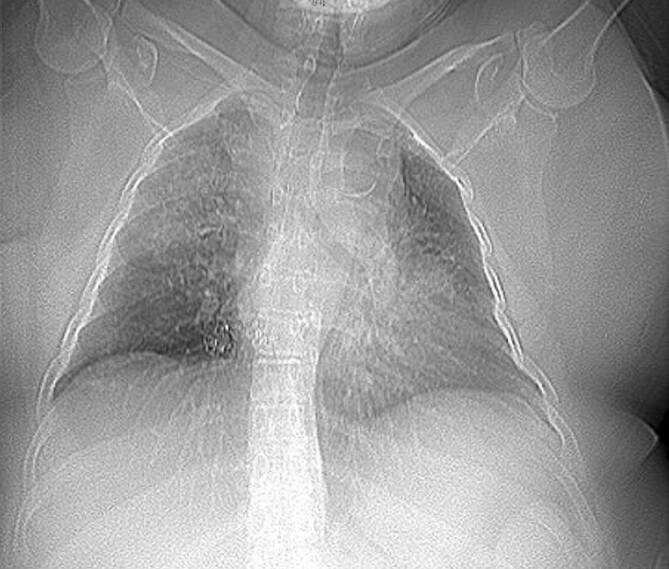

Im Fall 1 einer 57-jährigen Patientin wurde die Indikation zur frühen TS in dem Zeitintervall einer verhältnismäßig hohen Erkrankungsaktivität gestellt. Bei der Patientin mit einem BMI von >30 kg/m^2^ war eine prolongierte Weaningphase zu erwarten (Abb. 4). Durch den ET bei Beatmung in Bauchlage zeigten sich trotz präventiver Maßnahmen sehr frühzeitig Druckulzera der Mundwinkel beidseits und zudem Schwellungen der Zunge. In dieser Situation waren aufgrund der Adipositas und der Zungenschwellung weder eine extrathorakale noch transösophageale Echokardiographie zur Beurteilung der Herzfunktion möglich. Nach TS war eine Beatmung in Bauchlage problemlos möglich.

Bei 3 weiteren Patienten (Fall 2, 4, 5) wurde aufgrund einer Langzeitintubation mit prolongiertem Weaning (mindestens 3 Spontanatmungsversuche) die TS indiziert.

**Figure 5:**
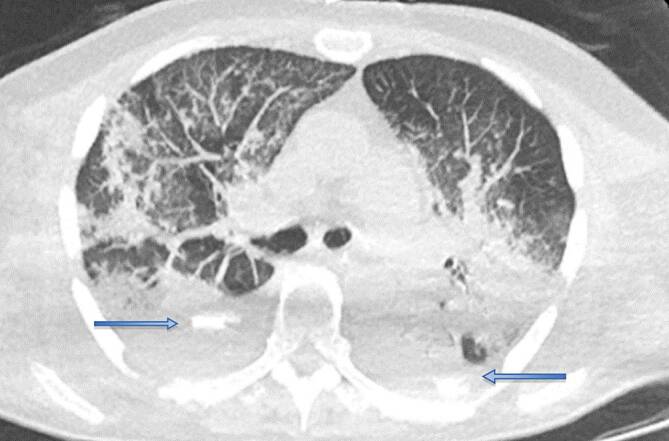

Im Fall 3 eines bereits dialysepflichtigen und mit einer ECMO versorgten Patienten kam es wiederholt während der invasiven Beatmung lageabhängig zu einer unkontrollierten Leckage des Beatmungssystems (Abb. 5). Um das Risiko einer virushaltigen Aerosolkontamination durch Umlagerungen so gering wie möglich zu halten, wurde abweichend von der SOP interdisziplinär entschieden, die operative TS am Patientenbett auf ITS durchzuführen. Präoperativ erhielt der Patient zur Gerinnungsoptimierung Thrombozytenkonzentrate. Dieser ECMO versorgte Patient verstarb 14 Tage nach der TS infolge einer Candidasepsis und multiplen anderen Organschäden.

Zwei Patienten konnten nach erfolgreichem Weaning 1 (Fall 4) bzw. 3 (Fall 1) Woche/n nach TS in eine weiterbehandelnde Klinik bzw. Frührehabilitation verlegt werden. Trotz erfolgreichem Weaning nach TS ist bei 2 Patienten (Fall 2 und 5) 3 bzw. 4 Wochen postoperativ eine weitere intensivmedizinische Behandlung erforderlich (nicht SARS-CoV‑2 verursachte Erkrankungen, z. B. Zosterenzephalitis, akutes Nierenversagen). Tendenzen bezüglich verbesserter Weaningverläufe der Patienten nach TS sind aufgrund der geringen Fallzahl und der besonderen Fallkomorbiditäten nicht ableitbar.

Bei 4/5 TS war sowohl der intra- als auch postoperative Verlauf komplikationslos. Im Fall 2 musste 14 Tage postinterventionell eine Blutung infolge einer lokalen Wundheilungstörung operativ versorgt werden.

Es wurden 4/5 TS und die TS-Nachblutung von einem Operateur (eine OP durch anderen Operateur) und jeweils 3 verschiedenen Operationsassistenzen durchgeführt. In allen Fällen wurde ein erfahrenes und SOP-geschultes, aber personell nicht konstantes Anästhesieteam sowie anästhesiologisches und Operationspflegepersonal eingesetzt. Dies entspricht auch dem klinischen Alltag (Personal in Schichtsystemen), in dem die Versorgung sowohl geplanter als auch Notfalleingriffe an den Atemwegen jederzeit bei COVID-19-Patienten gewährleistet werden muss. Die konsequente Anwendung der SOP vermittelte allen Beteiligten subjektiv Sicherheit im Umgang mit der infektiologisch besonderen Situation. Aufgrund der im Fall 3 intraoperativ kurzzeitig aufgetretenen Beatmungsleckage bei Manipulation an der Trachea ohne Hinweis auf eine Cuff-Beschädigung, erfolgte 5 Tage postoperativ eine vorsorgliche Nasen-Rachen-Abstrichuntersuchung (SARS-COV‑2 PCR) der beteiligten Mitarbeiter. Im Rahmen eines Mitarbeiterscreenings wurden alle HNO-Operateure präoperativ im Nasen-Rachen-Abstrich auf SARS-CoV‑2 sowie SARS-CoV-2-IgG (ELISA) negativ getestet. Nach aktuellem Kenntnisstand haben sich infolge der TS bei dem medizinischen Personal 3 Wochen postoperativ weder Symptome gezeigt noch wurde im Fall einer Testung eine COVID-19-Infektion nachgewiesen.

Die Deutsche Gesellschaft für Anästhesiologie und Intensivmedizin (DGAI) empfiehlt in ihren S3 (2017) bzw. S2k-Leitlinien (2019) eine TS für Patienten mit der dauerhaften Notwendigkeit einer invasiven Beatmung und fehlenden Optionen zur nichtinvasiven Ventilation (NIV) sowie bei allen anderen intubierten, invasiv beatmeten Patienten mit klinisch beobachteten prolongierten Weaning oder Beatmung länger als 7 Tage nach den ersten erfolglosen Spontanatmungsversuchen („spontaneous breathing trial“, SBT) zur Reduktion der, mit der endotrachealen Intubation assoziierten Komplikationen (tracheale und laryngeale Schädigungen und erhöhtes Infektionsrisiko der unteren Atemwege; [1, 6, 26, 29]). Weitere Argumente für die TS sind die Verkürzung der Dauer einer nachteiligen Sedierung, die zur Toleranzsteigerung bei liegendem endotrachealen Tubus erforderlich ist, und damit eine Vermeidung der sonst erforderlichen „On-/off-Situation“ durch Intubation/Extubation mit einer zusätzlich möglichen Erhöhung der Sterblichkeit nach einem Extubationsversagen mit erforderlicher Reintubation [31]. Zusätzlich werden auch SBT erleichtert und damit die Beatmungszeit verkürzt. Hierbei gilt es, den Zeitpunkt der TS individuell zu wählen [2]. In Abhängigkeit von der Latenz zur Intubation werden frühe (<7 Tage) von späten (ab 8–10 Tagen) TS unterschieden [29]. Frühe TS werden in den Leitlinien mit hoher Evidenz nicht empfohlen [1]. Ausgenommen davon sind TS aufgrund dauerhafter Notwendigkeit einer TS.

In der zweiten Version der S1-Leitline „Empfehlungen der intensivmedizinischen Therapie von Patienten mit Covid 19“ der DGAI wird die Indikation und Methode zur Durchführung einer TS im Sinne einer Einzelfallentscheidung empfohlen und berücksichtigt den speziellen Umgang des Weanings bei COVID-19-Patienten nicht [34].

International wird eine prolongierte invasive mechanische Beatmung bei COVID-19-Patienten, von bis zu 21 Tagen und länger propagiert, mit dem Ziel, möglichst einen mehrfach negativen SARS-CoV-2-Abstrich zu erreichen [12, 14, 38]. Ein Virusnachweis aus dem Respirationstrakt war bei ITS-Patienten sogar bis 31 Tage nach Symptombeginn möglich [9]. Grundsätzlich müssen aus unserer Erfahrung derzeit bei jeder TS im Verlauf einer COVID-19-Erkrankung trotz einmalig negativen SARS-CoV-2-Nachweises noch eine potenzielle Viruslast angenommen und deshalb die erforderlichen hygienischen Erfordernisse beachtet werden.

Aufgrund fehlender Prognoseparameter für die Beatmungsdauer von COVID-19-Patienten kann es momentan keine Empfehlung für den optimalen Zeitpunkt einer TS geben. Zudem stehen erfolgversprechende medikamentöse Therapien der Erkrankung und die abschließende Bewertung von Antikörperuntersuchungen aus. Somit wird die Anlage eines plastischen TS bis auf weiteres eine interdisziplinäre Einzelfallentscheidung bleiben [21]. In manchen Fällen sind aufgrund der erschwerten respiratorischen Situation mit permanentem Lagewechsel bereits zu einem frühen, d. h. sehr aktiven Zeitpunkt der Erkrankung eine TS erforderlich. Auch die Beatmung in Bauchlage ist mit TK möglich. Die Vorteile der TS bei Langzeitbeatmung in der prolongierten Weaningphase durch Verkürzung der täglichen Aufwachphasen für SBT, verminderten Sedierungsbedarf ohne ET, Reduktion des Totraumvolumens, des Atemwegwiderstandes und der damit verbundenen Atemarbeit [29] überwiegen auch für COVID-19-Patienten. Bei hohem Patientenaufkommen können dadurch möglicherweise zeitnaher wieder freie Respiratorplätze für neue Patienten zur Verfügung stehen. Zum Schutz des Personals vor nosokomialen Infektionen sollte der Zeitraum einer geringeren COVID-19-Erkrankungsaktivität bei der Wahl des Zeitpunktes der TS situationsangepasst Berücksichtigung finden.

Die Durchführung einer TS bei COVID-19-Erkrankten im interdisziplinären Kontext und mit Unterstützung der Abläufe durch eine klinikinterne SOP und deren Training, ermöglicht einen sicheren Ablauf für Patient und das medizinisches Personal. Insbesondere eine SOP-gestützte unmissverständliche Kommunikation (Abb. 2) kann eine akzidentielle unkontrollierte Aerosolbildung, wie bei Botti et al. beschrieben, verhindern [7].

Auch bei Beachtung aller Schutzmaßnahmen sind Restrisiken für das Personal jedoch nie vollständig ausgeschlossen. Deshalb sind eine engmaschige Symptomselbstkontrolle und Testungen des Personals auf SARS-COV‑2 im Nasen-Rachen-Abstrich bei Auftreten von Symptomen empfehlenswert, um frühzeitig Infektionen zu detektieren.

Aus eigener Erfahrung sehen wir klinikintern Vorteile für die Durchführung einer TS im Operationssaal, da er optimale räumliche als auch interventionelle Voraussetzungen für diesen Eingriff unter besonderem Personalschutz bietet. Insbesondere bei hoher Auslastung der Intensivstationen führt eine Intervention am Patientenbett zu zusätzlicher ITS-Personalbindung bei beschränkter räumlicher Kapazität (ECMO, Dialyseeinheit) sowohl für das Operationsteam als auch das Operationsequipment (steriler Operationstisch, Sauger, bipolare Koagulation, Lichtquelle). Für eine TS von COVID-19-Patienten muss sowohl im Operationssaal als auch auf der ITS insbesondere aufgrund der Schutzmaßnahmen ein gesteigerter Zeitaufwand eingeplant werden.

Grundsätzlich sollten *Notfalltracheostomien mit unbekanntem COVID-Status *mit allen hier genannten vorsorgenden Schutzmaßnahmen erfolgen. Bei *elektiven Tracheostomien *wird zusätzlich eine präoperative PCR-Testung auf SARS-COV‑2 und anschließende stationäre Quarantäne mit Mund-Nasen-Schutz bis zum Eingriff empfohlen.

## Fazit für die Praxis

- Die Indikationsstellung und der Zeitpunkt einer Tracheostomie (TS) bei COVID-19-Patienten sind immer interdisziplinäre Entscheidungen.
- Logistische und personelle Vorbereitungen vor einer TS von COVID-19-Patienten sind essenziell zur Vermeidung von Infektionen des medizinischen Personals insbesondere in Notfallsituationen.
- Unabdingbar ist eine permanente Kommunikation des Operationsteams über den Operationsfortschritt sowie anästhesiologischer bzw. intraoperativer Probleme.
- Klinikinterne interdisziplinäre SOPs (Standard Operation Procedure) ermöglichen ein strukturelles Vorgehen, eine Feedbackkontrolle und nach konsequenter Prüfung eine Adaptation derselben.
